# Green Tea Polyphenol Epigallocatechin-3-Gallate-Stearate Inhibits the Growth of *Streptococcus mutans*: A Promising New Approach in Caries Prevention

**DOI:** 10.3390/dj6030038

**Published:** 2018-08-06

**Authors:** Amy Lynn Melok, Lee H. Lee, Siti Ayuni Mohamed Yussof, Tinchun Chu

**Affiliations:** 1 Department of Biology, Montclair State University, Montclair, NJ 07043, USA; meloka1@mail.montclair.edu (A.L.M.); leel@montclair.edu (L.H.L.); mohamedyusss@montclair.edu (S.A.M.Y.); 2 Department of Biological Sciences, Seton Hall University, South Orange, NJ 07079, USA

**Keywords:** epigallocatechin-3-gallate-stearate, *Streptococcus mutans*, biofilm, colony forming assay

## Abstract

*Streptococcus mutans* (*S. mutans*) is the main etiological bacteria present in the oral cavity that leads to dental caries. All of the *S. mutans* in the oral cavity form biofilms that adhere to the surfaces of teeth. Dental caries are infections facilitated by the development of biofilm. An esterified derivative of epigallocatechin-3-gallate (EGCG), epigallocatechin-3-gallate-stearate (EGCG-S), was used in this study to assess its ability to inhibit the growth and biofilm formation of *S. mutans*. The effect of EGCG-S on bacterial growth was evaluated with colony forming units (CFU) and log reduction; biofilm formation was qualitatively determined by Congo red assay, and quantitatively determined by crystal violet assay, fluorescence-based LIVE/DEAD assays to study the cell viability, and scanning electron microscopy (SEM) was used to evaluate the morphological changes. The results indicated that EGCG-S was able to completely inhibit growth and biofilm formation at concentrations of 250 µg/mL. Its effectiveness was also compared with a commonly prescribed mouthwash in the United States, chlorhexidine gluconate. EGCG-S was shown to be equally effective in reducing *S. mutans* growth as chlorhexidine gluconate. In conclusion, EGCG-S is potentially an anticariogenic agent by reducing bacterial presence in the oral cavity.

## 1. Introduction

Dental caries, or tooth decay, is a multifactorial disease that affects a large percentage of today’s society [1,2]. Of the thousands of resident bacteria present in the oral cavity, they maintain a relatively neutral pH of around 6.8 [3]. Problems arise when this pH drops to a more acidic value, which promotes the demineralization of the enamel resulting in dental caries.

While it is obvious that dental caries are extremely problematic in underdeveloped and underprivileged areas, this disease is also seen extensively among privileged societies [4,5]. Dental caries pathogenesis involves several steps including the formation of a biofilm. A biofilm is defined as a community of bacteria that attach to a surface. While dental plaque is moderately specialized, it still shares the main properties of all biofilms. Biofilm formation is a three-stage process: docking, locking, and maturation [6,7]. *S. mutans* often gets the most attention in dental related studies because it has been previously shown to favor attachment to tooth enamel [8,9].

A popular drink around the world, tea is made from the infusion of dried *Camellia sinensis* leaves. Eastern cultures, such as China and Japan, are known to use tea medicinally based on its many health benefits. Previous studies have established that *Camellia sinensis*, especially the non-fermented type commonly known as green tea, has numerous medicinal advantages. These preceding studies have recognized green tea to have anti-inflammatory, antiviral, antifungal, antioxidant, protein-denaturing, anti-mutagenic, anti-diabetic, anticarcinogenic, and antibacterial characteristics [10,11,12,13,14,15,16,17,18,19]. The remedial effects of green tea are thought to be a result of the polyphenolic catechins present in green tea. The most active catechin, epigallocatechin-3-gallate (EGCG), makes up most of the content of the catechins at 59% [20]. However, several studies indicated that EGCG is unstable and less bioavailable [21,22,23,24]. A modified lipophilic derivative of EGCG called epigallocatechin-3-gallate-stearate (EGCG-S) has been synthesized with better stability and improved bioavailability [24]. Because these green tea components are known to have antibacterial activity, it has been shown that these bioactive components are also anticariogenic. Dental research has been completed in vivo in both animal and human participants demonstrating that green tea reduces carious incidents [15,25]. Previous literature reported that green tea extracts have short-term anti-plaque capabilities [26].

In order to determine EGCG-S’s effect on *S. mutans*, both qualitative and quantitative analyses were performed to observe its effect on growth inhibition and biofilm reduction. Furthermore, EGCG-S was compared with chlorhexidine gluconate, a common prescription for dental infections.

## 2. Materials and Methods

### 2.1. Culturing and Maintenance of Bacterial Cultures

*Streptococcus mutans* (*S. mutans*) Clarke was purchased from ATCC (ATCC^®^ 25175^TM^) and maintained at 37 °C aerobically with consistent shaking at 250 rpm. All cultures were maintained in nutrient agar (Difco^TM^, Detroit, MI, USA) or nutrient broth (Difco^TM^, Detroit, MI, USA). Fresh overnight cultures were used for each experiment. The purity of the culture was checked periodically.

### 2.2. Preparation of EGCG-S

EGCG-S was purchased from Camellix LLC, Augusta, GA, USA. EGCG-S was prepared using ethanol. Stock concentrations (5 mg/mL or 2.5 mg/mL) were prepared and diluted to the required concentrations needed for each experiment. The media containing ethanol were used as negative controls according to the EGCG-S concentration.

### 2.3. Colony Forming Unit (CFU) Assay

Each culture was treated with 0, 100, 200 and 250 µg/mL of EGCG-S respectively and was incubated at 37 °C for 1 h. These samples were serial diluted (from 10^−2^ to 10^−8^) and 100 µL of each dilution was spread onto nutrient agar plates aseptically and incubated overnight at 37 °C. All experiments were carried out in triplicates. Colony forming units (CFU) were recorded and the percentage of inhibition was calculated as follows:

% of Inhibition = [(CFUcontrol − CFUtreated)/CFUcontrol] × 100(1)

### 2.4. Viability Assay

The LIVE/DEAD^®^ BacLight™ Bacterial Viability Kit (Thermo Fisher, Catalog number: L7007) was used according to the manufacture recommendation. Molecular probes used in this kit include SYTO 9, green fluorescent dye stains intact cell membrane, and propidium iodide (PI), red fluorescence dye for damaged cell membrane. All samples were viewed under a fluorescent microscope (ZEISS Axio Scope A1).

### 2.5. Congo Red Assay

Congo red (Sigma-Aldrich C6767) agar was prepared according to the procedure outlined by Schwartz [27]. Positive, negative controls and EGCG-S (50, 100, 200 and 250 µg/mL) treated cultures were placed onto the respective wells and the plates were observed every day over a 4-day period. Black precipitation on the red agar indicates positive results for biofilm formation. All assays were done in triplicates.

### 2.6. Crystal Violet Assay

The cultures were treated with 100, 200, and 250 µg/mL of EGCG-S respectively and allowed to incubate at 37 °C for 4 days. The plates were aspirated, washed with 1X PBS and stained with 0.1% crystal violet for 30 min. The crystal violet was then aspirated, washed, and the plates were inverted until completely dry. One milliliter of 30% acetic acid was added into each well. OD readings were taken at 595 nm [28]. All experiments were done in triplicates with mean and standard deviation calculated. These readings were then used to determine the percentage of biofilm inhibition.

% of Inhibition = [(ODcontrol − ODtreated)/ODcontrol] × 100(2)

### 2.7. Scanning Electron Microscopy (SEM)

A sterile coverslip was placed at the bottom of each well in 6-well plates. Overnight cultures with or without EGCG-S 250 µg/mL were pipetted into the well and allowed to incubate at 37 °C for 4 days. Samples were prepared according to the procedure reported previously [29]. Finally, the samples were mounted onto a stub and coated with a thin layer of metal film using the Denton IV Sputter Coater before microscopic observation.

### 2.8. Time Course Study

This study is to determine different treatment times of EGCG-S and chlorhexidine gluconate on *S. mutans*. The times selected for this study were 5 s, 30 s, 1 min, and 5 min. One mL of overnight culture was centrifuged and the pellet was then suspended in either EGCG-S (250 µg/mL), or chlorhexidine gluconate (0.1%). At each time point, serial dilutions were made, 100 µL of the sample was retrieved and plated onto nutrient agar plates. Cultures suspended in nutrient agar were used as controls. All plates were incubated at 37 °C overnight and CFUs were determined.

### 2.9. Statistical Analysis

All assays were performed in triplicates and the data analyzed using one-way Analysis of Variance (ANOVA) with Dunnett’s Post Hoc Test using SPSS Version 20.0 (IBM Corp. Armonk, NY, USA).

## 3. Results

### 3.1. The Effect of EGCG-S on S. mutans

The effect of different concentrations EGCG-S on the growth of *S. mutans* was monitored using a colony forming unit (CFU) assay. No inhibition of *S. mutans* in all negative controls were observed. Log reduction was calculated from the results obtained from the CFU assay (Table 1). Compared with control, log reduction was 1.19 ± 0.02 when cells were treated with 100 µg/mL EGCG-S; 2.04 ± 0.02 at 200 µg/mL EGCG-S; and 2.65 ± 0.01 at 250 µg/mL EGCG-S.

**Figure 1:**
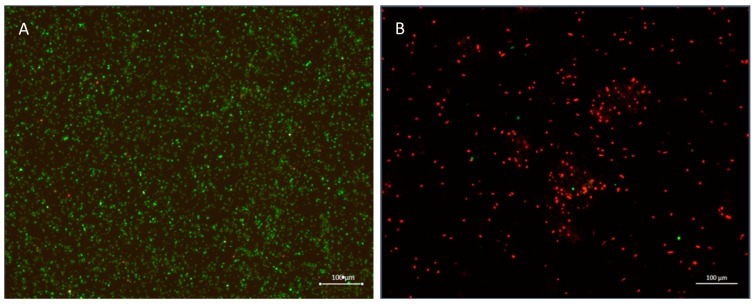
Cell viability assay. (**A**) Control (untreated *S. mutans*). Cells fluorescent green are viable cells. (**B**) *S. mutans* treated with EGCG-S 250 µg/mL for 1 h (430X). Cells fluorescent red indicated dead cells.

Fluorescent microscopy was used to evaluate cell viability with BacLight™ Bacterial Viability Kit. Cell viability was assessed before and after treatment with 250 µg/mL EGCG-S as shown in Figure 1. The control group was shown to have a high population density and fluoresced the green color (Figure 1A), indicating that most of the population was alive and viable. After treatment with 250 µg/mL EGCG-S, nearly the entire population fluoresced red indicating that most cells were not viable post-treatment (Figure 1B).

### 3.2. The Effect of EGCG-S on Biofilm of S. mutans

**Figure 2:**
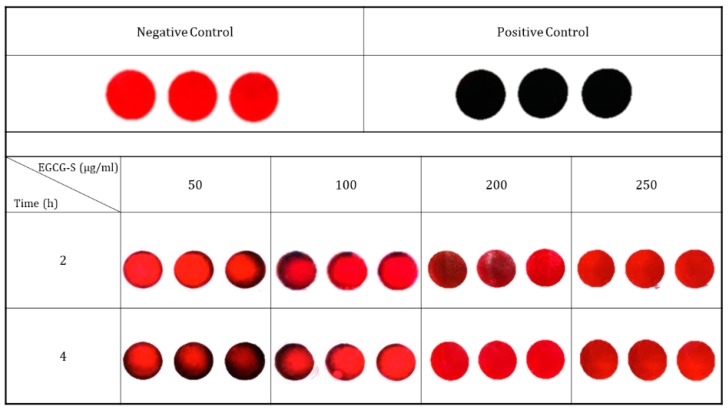
Congo red biofilm assay. Negative controls are represented by a red color and signify no biofilm growth. Positive controls are represented by a dark color and signify that biofilm growth has occurred. At concentrations of 200 and 250 µg/mL biofilm formation was inhibited completely.

EGCG-S at 250 μg/mL was able to inhibit the growth of cells. In this study, Congo red agar was used to qualitatively examine the effects of EGCG-S on biofilm formation. The results of Congo red analysis are shown in Figure 2. Results with a black color on agar, as shown, are Positive Control; Negative Control, with a red color, represents no biofilm formation. It demonstrated that for samples treated with 50 µg/mL and 100 µg/mL of EGCG-S for 2 and 4 h treatment, the biofilm was significantly reduced but not completely inhibited, as it appeared as partially black. When treated with 200 µg/mL the biofilm was nearly completely inhibited at 2 h treatment and completely inhibited at 4 h treatment. With the concentration of 250 µg/mL at 2 h and 4 h, biofilm formation was completely inhibited. These results indicated that EGCG-S could completely inhibit biofilm formation of *S. mutans* at 200 µg/mL for 4 h and 250 µg/mL for 2 to 4 h. Lower concentrations were not able to completely inhibit the formation of biofilm.

**Figure 3:**
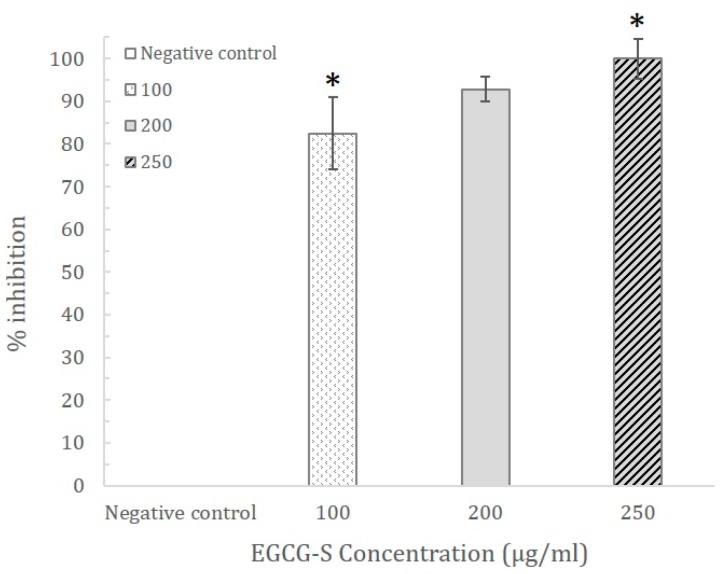
The percentage of inhibition of EGCG-S treated samples vs. control from the Crystal Violet Assay. EGCG-S demonstrates an excellent inhibitory effect by having 100% inhibition at the concentration of 250 µg/mL. Results were shown with mean and standard deviation (*n* = 3). Data analysis indicated significant difference between 100 and 250 µg/mL EGCG-S (with asterisks).

In order to quantitatively study the effect of EGCG-S on biofilm formation, the crystal violet assay was carried out. The results exhibited that 100, 200, and 250 µg/mL of EGCG-S were able to inhibit biofilm formation by 82.49 ± 8.50%, 92.75 ± 2.9%, and 100 ± 4.7%, respectively, as shown in Figure 3. The concentration of 250 µg/mL EGCG-S was able to completely inhibit biofilm formation from occurring, which further supported the results from Congo red analysis. Although no biofilm dark precipitation was observed at a concentration of 200 µg/mL (92.75%), the complete inhibition was determined to be 250 µg/mL.

**Figure 4:**
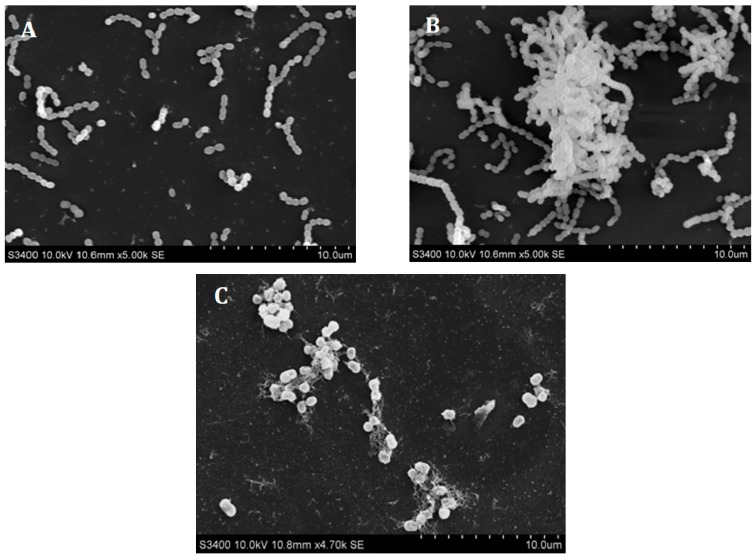
Scanning electron microscopy (SEM) of *S. mutans*. (**A**) Control *S. mutans* cells; (**B**) Untreated *S. mutans* cells were grown for 4 days; (**C**) *S. mutans* cells treated with 250 µg/mL EGCG-S for 4 days.

Scanning electron microscopy (SEM) images were taken before and after treatment with 250 µg/mL EGCG-S. The control of untreated cells confirmed the morphology of *S. mutans* (Figure 4A) and after 4 days, biofilm was observed (Figure 4B)*.* After 4 days of 250 µg/mL EGCG-S treatment, the morphology of the cells was altered suggested the integrity of the cells was damaged. There is no biofilm observed as shown in Figure 4C.

### 3.3. Time Course Study of S. mutans treated with 250 µg/mL EGCG-S and Chlorhexidine Gluconate

**Figure 5:**
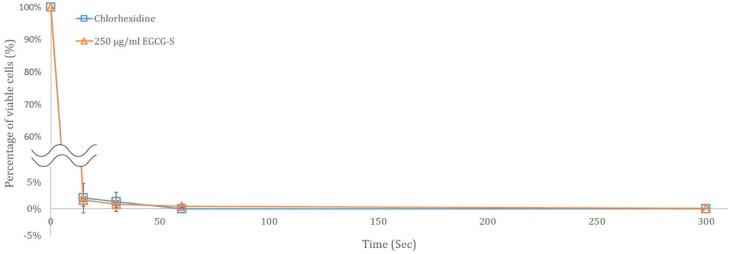
Time course study of 250 µg/mL EGCG-S and 0.1% chlorhexidine gluconate on the growth of *S. mutans*.

For evaluating if EGCG-S can be a potential organic mouthwash, the short term time course study was carried out for 0 to 5 min to determine the minimum time needed to inhibit *S. mutans*. In this study, 0 s, 15 s, 30 s, 1 min, and 5 min were used, CFU was determined and the percentage of viability was calculated. Untreated bacteria were used as the control. A parallel study using 0.1% chlorhexidine gluconate was also carried out to compare their effects. The results are shown in Figure 5 and clearly indicated that by 1 min, both EGCG-S and chlorhexidine gluconate were able to completely inhibit the growth of the cells.

## 4. Discussion

This is the first study investigating EGCG-S as a potential anticariogentic agent. In this study, it suggested that EGCG-S can inhibit the growth of *S. mutans.* The Congo red assay provided preliminary information for the exposure time and concentrations necessary for EGCG-S on their effect on biofilm formation and indicate the presence/absence of biofilm. The Crystal Violet (CV) assay showed that 250 µg/mL EGCG-S was able to completely inhibit biofilm formation. Both experiments confirmed that EGCG-S was able to reduce bacterial growth and biofilm formation in a dose-dependent manner. 

While the mechanism of EGCG-S is not yet fully understood, both the fluorescence microscopy and scanning electron microscopy results displayed a possible association to cell surface integrity. This suggested the possible mechanisms of EGCG-S may be similar to one of the mechanisms of EGCG that have been reported previously to damage the cell membrane [30,31,32,33,34,35,36], or cell wall [37,38,39] or interfere with polysaccharides interaction [6]. Molecular research should be carried out to further elucidate the mechanism of EGCG-S on *S. mutans* and other biofilm forming bacteria.

It is common for patients with infections of the oral cavity to be prescribed with 0.1% chlorhexidine gluconate. This comparative study was conducted over a period of 5 min and EGCG-S was effective in reducing bacterial growth at 1 min similar to the prescribed mouth wash chlorhexidine gluconate.

## Figures and Tables

**Table 1:** Colony forming units (CFU) (cells/mL) and log reduction of epigallocatechin-3-gallate-stearate (EGCG-S) treated samples.

EGCG-S Concentration (µg/mL)	CFU (cells/mL)	Log Reduction
0	1.01 × 10^12^ ± 9.24 × 10^10^	0
100	6.57 × 10^10^ ± 3.20 × 10^9^	1.19 ± 0.02
200	9.20 × 10^9^ ± 4.00 × 10^8^	2.04 ± 0.02
250	2.30 × 10^9^ ± 2.65 × 10^8^	2.65 ± 0.01
